# The Use of High Volume Plasmapheresis in Acute Liver Failure

**DOI:** 10.7759/cureus.8721

**Published:** 2020-06-20

**Authors:** Landon Tam, Constantine Karvellas, Eric Sy

**Affiliations:** 1 Internal Medicine, University of Saskatchewan, Regina, CAN; 2 Critical Care Medicine, University of Alberta, Edmonton, CAN; 3 Critical Care Medicine, University of Saskatchewan, Regina, CAN

**Keywords:** plasmapheresis, hepatology, adult gastroenterology

## Abstract

High volume plasmapheresis (HVP) is defined as an exchange of 8-12 L or 15% of ideal body weight with fresh-frozen plasma. It has been reported that HVP can improve outcomes in patients with acute liver failure (ALF) and/or acute-on-chronic liver failure (ACLF). Here, we present a case of a 34-year-old man presenting with ALF that led to multi-organ failure who received HVP in the intensive care unit that improved his biochemical parameters, volume status, and hemodynamics. However, despite objective clinical and biochemical improvements, the patient had developed signs of potential brain injury, and subsequently the family withdrew care. This case and the associated literature review highlight the potential value of HVP in facilities who do not have access to liver transplantation or other means of extracorporeal liver support systems.

## Introduction

Acute liver failure (ALF) can result in irreversible shock, cerebral herniation, or the development of multi-organ failure (MOF) [1]. Common etiologies of ALF include drug or toxin exposure, viral infections, autoimmune disease, and ischemia. The management strategy of ALF patients includes treatment of underlying cause when possible and preservation of vital organ function to limit progression to MOF [2]. Some patients may have progressive deterioration despite these measures, and may require liver transplantation. Many liver transplant centers have used extracorporeal liver support systems (ECLS), such as molecular adsorbent recirculating system (MARS) therapy, to support patients with ALF or acute-on-chronic liver failure (ACLF), and reduce their overall mortality. However, not all centers have access to liver transplantation or ECLS. One randomized study and a separate case series have demonstrated the safety and potential improvement in transplant-free survival with the use of high volume plasmapheresis (HVP) [1,3,4]. HVP is defined as an exchange of 8-12 L or 15% of ideal body weight with fresh-frozen plasma [1]. In ALF, cytokines are largely responsible for the progression of MOF and HVP removes these cytokines from the systemic circulation [1,5]. We report a case of implementing HVP in an adult with ALF in the intensive care unit (ICU) at a tertiary care center.

## Case presentation

A 34-year-old male with known alcohol use disorder, alcohol withdrawal seizures, and atrial fibrillation presented to the emergency room (ER) with a history of symptoms related to alcohol withdrawal and suicidal ideations for the past day. On presentation, he claimed that he had been abstinent from alcohol for two to three days, but previously, he was drinking 40 ounces of alcohol per day for the last six months. In addition, he stated that he was having thoughts of self-harm, but he denied any drug ingestion prior to presentation. On examination, his vitals were all within normal limits, and his Glasgow Coma Scale (GCS) score was 15/15. He appeared agitated with bilateral postural hand tremors and had diffuse abdominal tenderness on palpation. The rest of his physical examination was unremarkable, and he had no signs or stigmata of chronic liver disease. His laboratory investigations included hemoglobin, 119 g/L; white blood cells, 6.3 x 10^9^/L; platelets, 61 x 10^9^/L; serum sodium, 125 mmol/L; creatinine, 106 μmol/L; anion gap, 18 mmol/L; alanine aminotransferase (ALT), 613 units/L; alkaline phosphatase (ALP), 79 units/L; total bilirubin, 66 μmol/L; direct bilirubin, 46 μmol/L; and albumin level, 33 g/L. He did not have a previous ammonia level measurement done in the past to discern a baseline. His liver biochemistry was initially attributed to alcoholic hepatitis, and his clinical symptoms were attributed to alcohol withdrawal. Unfortunately, a toxicology panel was not done in the ER. The patient was subsequently admitted to the non-teaching General Internal Medicine service by the ER physician, with further workup and management to be done the following morning. However, within six hours of admission, the patient progressively became more agitated with fluctuating level of consciousness (LOC) and eventually developed a one-minute tonic-clonic seizure that was followed by a progressive decrease in LOC requiring intubation. He became quite hypotensive despite appropriate fluid resuscitation, and required high doses of vasopressors. The patient was subsequently transferred to the ICU.

**Figure 1:**
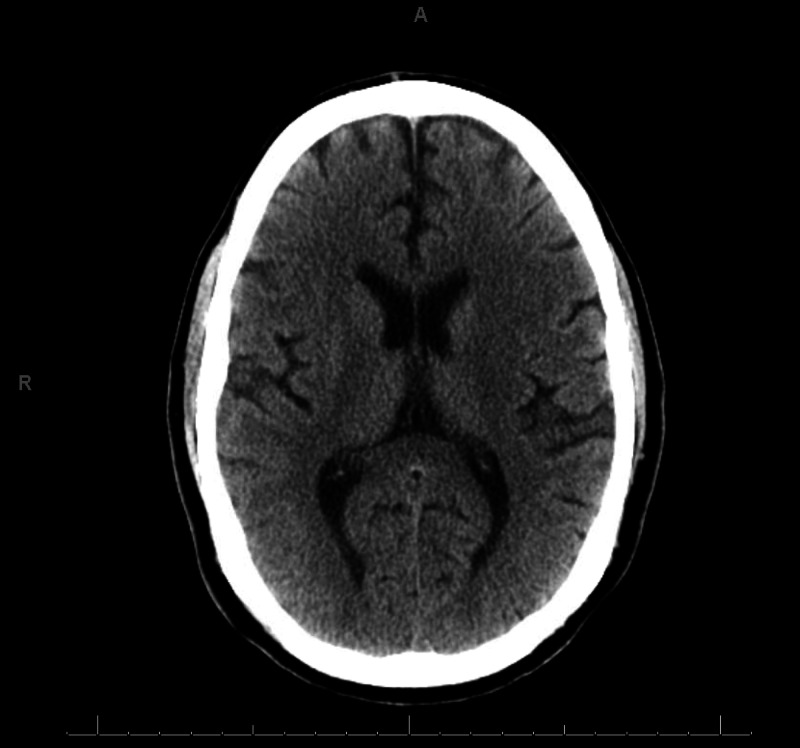
Head CT of the Patient Non-contrast CT of the brain demonstrates no evidence of intracranial pathology that would suggest a structural cause for the patient's decrease level of consciousness.

In the ICU, the patient was found to have worsening transaminitis in which his ALT had risen to 2,000 units/L and his aspartate aminotransferase (AST) peaked to nearly 13,000 units/L. He was also found to be in a profound metabolic acidosis with a lactate of 9.6 mmol/L. His international normalized ratio (INR) went up to 5.6 and his bilirubin peaked to 106 μmol/L. Furthermore, his LOC did not improve post-seizure, and his GCS score remained a 3/15 despite minimal sedation, with an associated arterial ammonia level of 316 μmol/L. He had mildly unequal and weakly reactive pupils. A CT scan did not demonstrate any cerebral edema (Figure 1). A toxicology panel demonstrated an acetaminophen level <20 μmol/L, an undetectable salicylates level, and the presence of benzodiazepines and tetrahydrocannabinols in the urine. The patient’s ethylene glycol level was interestingly elevated at 2 mmol/L, which may have suggested some form of co-ingestion. The osmolal gap was normal (<10 mOsm/kg) and his anion gap was 16 mmol/L at the time of the ethylene glycol measurement. Lastly, a complete hepatitis workup, which included autoimmune, metabolic, viral, and vascular causes, was also sent and was unremarkable. 

The etiology of his biochemical liver abnormalities was unclear at presentation. The differential diagnosis of his presentation included ALF possibly secondary to unknown ingestion or late presentation of acetaminophen overdose, ischemic hepatopathy secondary to shock of unknown etiology, and ACLF with possible ischemic or toxic precipitant. An ultrasound done the following day demonstrated fatty liver disease, without evidence of cirrhosis or portal hypertension. Thus, a presumptive diagnosis of ALF was made, although ACLF could not be definitively ruled out. The patient was empirically treated with N-acetylcysteine, thiamine, folic acid, pyridoxine, fomepizole, and continuous renal replacement therapy (CRRT). The premise to initiate CRRT was on the basis of managing the patient’s renal failure in the setting of volume overload, metabolic acidosis, and possible toxic alcohol ingestion. He was started on empiric antibiotics, although none of his cultures had grown any organisms during his hospital stay.

Given his worsening state, there was a discussion to transfer this patient to a regional transplant center for consideration of either transplant or MARS therapy. However, due to his known history and recent use of alcohol, he was not a candidate for transplant, and he was too hemodynamically unstable for transport. As a result, they recommended a trial of HVP.

His first run of HVP was done at the end of day 2 of his ICU stay in which 9 L of fresh-frozen plasma was exchanged, and the following morning the patient’s biochemical parameters such as his liver enzymes, albumin, INR, and bilirubin had all improved. Similarly, his vasopressor and ventilating pressures and his bladder pressure decreased (Table 1).

**Table 1:** Pre- and Post-HVP ALT: alanine aminotransferase; AST: aspartate aminotransferase; ALP: alkaline phosphatase; GGT: gamma-glutamyl transferase; INR: international normalized ratio; PEEP: positive end-expiratory pressure; PaO_2_/FiO_2_: partial pressure of arterial oxygen/fractional inspired oxygen; HVP: high volume plasmapheresis

Markers	Admission (lab work limited)	Pre	Post
ALT (IU/L)	613	1971	658
AST (IU/L)	N/A	12,936	6,183
ALP (IU/L)	79	85	55
GGT (IU/L)	N/A	795	239
Albumin (g/L)	33	22	28
Bilirubin (μmol/L)	66	93	33
INR	N/A	5.6	1.8
Ammonia (μmol/L)	N/A	316	108
Vasopressor support	None	Norepinephrine 2.0 mcg/kg/min, epinephrine 0.05 mcg/kg/min, and vasopressin 0.03 units/min	Norepinephrine 0.2-0.5 mcg/kg/min, epinephrine 0.02-0.05 mcg/kg/min
Ventilator settings	None	Peak 45 cm H_2_0, plateau 40-50 cm H_2_0	Peak 35-38 cm H_2_0, plateau 30-33 cm H_2_0
PEEP (cm H_2_O)	None	8	14
Driving pressure (cm H_2_O)	N/A	32-42	16-19
Tidal volume (mL)	N/A	530	480
PaO_2_/FiO_2_	N/A	163	180
pH	N/A	7.01	7.11
Bladder pressure (mmHg)	N/A	35	23
Encephalopathy (West Haven Criteria)	Grade 0	Grade 4	Grade 4

Despite improvements that morning on day 3 of his ICU stay, the patient’s LOC did not improve, and he showed signs of worsening pupil dilation and less reactivity. An electroencephalogram (EEG) was ordered that day, and it demonstrated generalized suppressed cerebral activity with no activity seen at two microvolt sensitivity, indicating a low likelihood of functional neurological recovery. Furthermore, prior to the next scheduled HVP that afternoon, the patient began to again develop worsening shock and ventilator support. Based on these changes in clinical condition, the patient’s next of kin made a decision to withdraw active medical management. A post-mortem examination was declined and not done.

## Discussion

In ALF, there is significant hepatocyte necrosis that is followed by the release of cytokines and adhesion molecules that results in a large-scale proinflammatory cascade [6]. This combination of proinflammatory cascade and hepatic dysfunction results in the build-up of toxins that cause systemic disturbances associated with ALF [7]. HVP technically falls under the umbrella term of artificial ECLS in which it replaces the patients' plasma with fresh-frozen plasma with the objective of detoxification and regulation of serum chemistry function [2,8]. By removing plasma cytokines, adhesion molecules and toxins while replacing plasma factors, HVP aims for the potential of modulating the immune system [1].

There has only been one study that demonstrated any statistical significance and efficacy for the use of any of the ECLS systems in ALF and that was with HVP [1,8]. In this prospective multicenter randomized control trial done by Larson et al. in 2016, 182 ALF patients were randomized to receive standard medical therapy (SMT) or SMT plus HVP for three days [1]. Biochemically, the patients’ bilirubin, INR, and ammonia levels all significantly decreased following HVP treatment, and they found that overall hospital survival was 58.7% for patients treated with HVP vs. 47.8% for SMT group alone. Therefore, they concluded benefits in transplant free survival with HVP administration. In a subgroup nested cohort study of 30 of these ALF patients in the same study, patients undergoing HVP were shown to have significantly reduced circulating levels of proinflammatory cytokines, with an associated decrease in neutrophil activation, further confirming the utility of HVP in combating the systemic inflammatory response associated with ALF [1]. In the aforementioned study, blood samples were taken prior to and after HVP, and this demonstrated a clear reduction in circulating levels of damage-associated molecular patterns (DAMPs), such as TNF-alpha, circulating histone-associated DNA, IL-4, IL-8, IL-10, L-selectin (CD62L), TGfb, and angiopoietin-2. All these molecules can be responsible for a proinflammatory cascade, and although this study did not show reduction in circulating monocytes and neutrophils, it demonstrated reduction of their derived production of proinflammatory mediators [1].

Presently, liver transplantation remains the only definitive management strategy for ALF patients for whom spontaneous liver recovery does not occur. However, many patients do not survive to liver transplant or they may not be candidates either for medical or psychosocial reasons, similar to this case [8]. As a result, this case demonstrates that there may be potential benefits of conducting HVP in centers without access to MARS or transplantation. However, HVP may improve certain biochemical parameters, but it may not improve the overall outcome as seen in this case. This suggests that prognosis may not improve once a certain threshold of MOF develops. Previous studies, including Larsen et al. in 2016 and case reports, initiated HVP in cases with diverse etiologies of liver failure and at various points in the disease course. Therefore, the timing of initiating HVP is still quite unclear. It is unknown at this time whether earlier intervention with HVP, such as before the development of overt encephalopathy, organ failure, or biochemical cutoffs may result in improved survival or potentially place the patient at increased risk of the complications of HVP.

## Conclusions

Lastly, very few studies have looked at HVP treatment in ALF due to specific etiologies. Further studies are warranted to assess the utility of HVP in specific etiologies of liver failure. For example, HVP has been reported to have positive effects on survival over MARS therapy for liver failure secondary to acetaminophen toxicity. Furthermore, cases and appropriate powered studies of liver failure requiring ECLS therapy are becoming increasingly difficult to find, given the scarcity of liver failure, efficiency to transplant, multicenter enrollment, and institutional SMT protocols. As a result, there is a great dependence on isolated care reports to guide treatment in those rare circumstances of liver failure without access to liver transplantation.
